# Challenging the workhorse: Comparative analysis of eukaryotic micro‐organisms for expressing monoclonal antibodies

**DOI:** 10.1002/bit.26951

**Published:** 2019-03-07

**Authors:** Hanxiao Jiang, Andrew A. Horwitz, Chapman Wright, Anna Tai, Elizabeth A. Znameroski, Yoseph Tsegaye, Hailley Warbington, Benjamin S. Bower, Christina Alves, Carl Co, Kanvasri Jonnalagadda, Darren Platt, Jessica M. Walter, Venkatesh Natarajan, Jeffrey A. Ubersax, Joel R. Cherry, J. Christopher Love

**Affiliations:** ^1^ Research and Development Amyris Inc. Emeryville California; ^2^ Engineering & Technology Biogen Cambridge Massachusetts

**Keywords:** alternate expression systems, antibody, genome engineering, recombinant protein expression, yeast expression systems

## Abstract

For commercial protein therapeutics, Chinese hamster ovary (CHO) cells have an established history of safety, proven capability to express a wide range of therapeutic proteins and high volumetric productivities. Expanding global markets for therapeutic proteins and increasing concerns for broadened access of these medicines has catalyzed consideration of alternative approaches to this platform. Reaching these objectives likely will require an order of magnitude increase in volumetric productivity and a corresponding reduction in the costs of manufacture. For CHO‐based manufacturing, achieving this combination of targeted improvements presents challenges. Based on a holistic analysis, the choice of host cells was identified as the single most influential factor for both increasing productivity and decreasing costs. Here we evaluated eight wild‐type eukaryotic micro‐organisms with prior histories of recombinant protein expression. The evaluation focused on assessing the potential of each host, and their corresponding phyla, with respect to key attributes relevant for manufacturing, namely (a) growth rates in industry‐relevant media, (b) adaptability to modern techniques for genome editing, and (c) initial characterization of product quality. These characterizations showed that multiple organisms may be suitable for production with appropriate engineering and development and highlighted that yeast in general present advantages for rapid genome engineering and development cycles.

## Graphical abstract

In this work, we present a comparative study of the potential of eight different eukaryotic microorganisms (yeasts, fungi, diatom, and protozoan) as alternative hosts for the expression of recombinant biopharmaceuticals. The study assesses the suitability of these hosts in a ‘head‐to‐head’ manner that would allow direct comparisons of their tractability for high‐density cultivation, engineering with advanced genome editing tools (CRISPR/Cas9), and the molecular attributes of recombinant antibodies expressed in the tractable hosts.

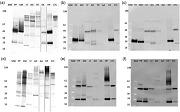

## 1 INTRODUCTION

Monoclonal antibodies represent a significant and growing class of drugs for the treatment of chronic diseases such as cancer, autoimmune diseases, neurodegenerative diseases, and cardiovascular diseases. This class of biopharmaceuticals also provides a promising route to both therapeutic and prophylactic treatments for infectious diseases such as HIV, malaria, tuberculosis, and gastroenteric diseases, among others. The global burden of diseases is expanding and the expected demand for certain protein therapeutics to treat large populations of affected patients may exceed the current capacity for production. For example, the use of an HIV‐neutralizing monoclonal antibody as a prophylactic has been estimated to require 1,000 kg (Ma et al., 2015) to 100 tons (Buyel, Twyman, & Fischer, 2017) of annual production to treat the at‐risk population in sub‐Saharan Africa, depending on dose. For perspective, an estimated 15.3 metric tons of monoclonal antibody products (all mammalian‐based) were produced in 2015, including Fc‐fusion proteins and antibody fragments (Levine & Cooney, 2017). Approvals of new monoclonal antibody products have ranged from 6 to 10 per year from 2015 to 2017, averaging over eight per year (data from FDA and EMA websites). Assuming this approval rate continues, the number of marketed antibody products will double by 2024 and triple by 2032 from the 71 in 2016 (Levine & Cooney, 2017).

Current production of commercial monoclonal antibodies for therapeutic purposes predominantly uses Chinese Hamster Ovary (CHO) cells. The average reported titer for commercial‐scale monoclonal antibodies in 2017 was 2.8 g/L (Langer & Rader, 2017). Advancements in cell line development and process intensification have moved batch culture productivity toward yields of 10 g/L and perfusion culture toward 2 g·L^−1^·day^−1^ for clinical stage monoclonal antibodies. However, further gains in productivity are likely to be incremental and fall short of levels needed to alleviate capacity concerns.

The capacity limitation is not the only consideration. The current cost of goods sold (COGS) is too high to consider therapies for the developing world (Sparrow, Friede, Sheikh, & Torvaldsen, 2017). Process intensification has achieved high titers by perfusion or concentrated fed‐batch cultivation, but the overall COGS remains in the range of $59 (perfusion)–$81 (concentrated fed‐batch) per gram of antibody—comparable with the average cost of those produced in a standard fed‐batch process ($71/g; S. Xu, Gavin, Jiang, & Chen, 2017). For the routine treatment of HIV or other infectious diseases, the cost of monoclonal antibodies must drop to $10</g (Hadley, 2013). A 6‐ to 10‐fold decrease in COGS will require significant changes in the manufacturing platform and will not result from incremental improvements in productivity or capital investments.

The marginal gains available for COGS, and for the productivity of processes that rely on CHO cells, has led to considerations on whether or not CHO cells represent the best host for future manufacturing platforms. Large‐scale production of industrial enzymes and other proteins have significantly lower COGS and could serve as models for achieving similar efficiencies with monoclonal antibodies (Singhania, Patel, & Pandey, 2010). Microbial hosts all provide potential benefits and utility for lowering production costs and for expressing glycosylated proteins such as monoclonal antibodies. Extensive glycosylation engineering has been performed in yeast, resulting in strains capable of producing fully humanized glycoproteins (Hamilton & Gerngross, 2007), including, monoclonal antibodies with effector functions (Li et al., 2006). However, the degree to which each potential host has been developed or rigorously assessed for use in biopharmaceutical manufacturing has varied significantly.

We previously outlined key characteristics for an ideal host to produce therapeutic proteins (Matthews et al., 2017). These included rapid growth coupled with established organelles and functions for protein processing and secretion. Toolkits for strain engineering are required to incorporate the recombinant protein into the host genome and to enable engineering of pathways for posttranslational modifications (e.g., glycosylation) and enhanced productivity (e.g., chaperones). A number of intrinsic attributes can also guide the selection of a potential host, including, demonstrated specific productivities, the lack of cosecreted proteases, the number of host cell proteins in the proteome and the potential for enhanced safety due to the lack of mammalian viral infectivity. Process‐related flexibility is also another consideration for reducing the costs of production. Examples are the use of lower cost or less refined feeds as well as robustness toward shear stress, pH, and temperature.

Eight eukaryotic micro‐organisms met all or most of these criteria and were selected for a comparative analysis to assess which ones may present suitable alternatives for further development to produce recombinant biopharmaceuticals. All eight have sequenced genomes and demonstrated the expression of complex heterologous proteins (Matthews et al., 2017). The methylotrophic yeasts *Pichia pastoris* (reclassified as *Komagataella phaffii* and *Komagataella pastoris*; Cereghino & Cregg, 2007) and *Hansenula polymorpha* (reclassified as *Ogataea polymorpha* and *Ogataea parapolymorpha*; Stockmann et al., 2009) were developed as expression systems with inducible promoters activated by a change in carbon source. *Kluyveromyces marxianus* has a long history of use in food fermentation and can grow efficiently on inexpensive lactose‐based media at a wide variety of temperatures (Raimondi et al., 2013). *Arxula adeninivorans* (reclassified as *Blastobotrys adeninivorans* ) is a dimorphic yeast that can grow on a wide variety of carbon and nitrogen sources (Stockmann et al., 2009). Filamentous fungi *Trichoderma reesei* (anamorph of *Hypocrea jecorina*; Singh et al., 2015) and *Aspergillus oryzae* (Fleissner & Dersch, 2010; Tsuchiya et al., 1994) are often used for homologous and heterologous enzyme production. The diatom *Phaeodactylum tricornutum* has been demonstrated capable of secreting fully assembled antibodies (Hempel & Maier, 2012). Several recombinant proteins have been produced in the protozoan *Leishmania tarentolae* and an expression system is available commercially (Basile & Peticca, 2009).

The intention of this study was to allow direct, parallel experiences in culturing each organism, manipulating their genomes to express therapeutic proteins, and characterizing the molecular attributes of the molecules they produced. We sought to establish whether one or more of these hosts might provide a suitable basis for further development broadly, and to provide a comparative framework to guide such development. For each species, three key parameters were evaluated: growth in industrially relevant media, engineerability of the host, and an initial assessment of product quality to establish a guideline for future development.

## 2 MATERIALS AND METHODS

### 2.1 Strains

The eight different host strains used in this study are listed in Table 1. All yeasts and filamentous host strains were obtained from USDA NRRL collection. *P. tricornutum* and *L. tarentolae* were purchased from UTEX (Austin, TX) and Jena Bioscience (Jena, Germany).

**Table 1:** Overview of eight selected hosts. Growth rates of all four yeast strains, *Leishmania tarentolae*, and *Pichia tricornutum* were measured in each host's regular growth media as described in Section 2.

Class	Species	Strain name	Genome (Mb) (References)	Number of chromosomes	Average growth rate (hr^−1^) (References)
Yeast	*Pichia pastoris*	NRRL Y‐48124	9.4 (Love et al., 2016)	4	0.40
	*Kluyveromyces marxianus*	NRRL Y‐7571	10.9 (Jeong et al., 2012)	8	0.57
	*Hansenula polymorpha*	NRRL Y‐7560	9.1 (Ravin et al., 2013)	7	0.38
	*Arxula adeninivorans*	NRRL Y‐17692	11.8 (Kunze et al., 2014)	4	0.40
Filamentous fungi	*Trichoderma reesei*	NRRL 15709	34.9 (Li et al., 2017)	7	0.202 (Tholudur, Ramirez, & McMillan, 1999)
	*Aspergillus oryzae*	NRRL 694	37 (Machida et al., 2005)	8	0.27 (Carlsen, Spohr, Nielsen, & Villadsen, 1996)
Other	*Leishmania tarentolae*	JENA T7‐TR	32.8 (Kazemi, 2011)	36	0.074
	*Phaeodactylum tricornutum*	UTEX 646	27.5 (Bowler et al., 2008; Maheswari, Mock, Armbrust, & Bowler, 2009)	34	0.013
Mammalian	CHO‐K1		2450 (X. Xu et al., 2011)	21	0.035

### 2.2 Construction of antibody expression cassettes

The anti‐CD20 antibody sequences provided by Biogen include heavy chain (HC) and light chain (LC) polypeptides. To drive secretion of the construct from the yeasts, the full‐length, 89 amino acid pre–pro‐α factor secretion leader from *Saccharomyces cerevisiae* (Waters, Evans, & Blobel, 1988) was added to the amino termini of both HC and LC. The amino acid sequences of anti‐CD20, Herceptin, and Rituxan were codon‐optimized according to host species preference using a codon optimization algorithm, and chemically synthesized by Gen9 (now Ginkgo Bioworks, Boston, MA), Twist (San Francisco, CA), or Integrated DNA technologies (IDT; San Diego, CA). DNA expression constructs were cloned in a variety of configurations under strong constitutive native and/or inducible promoters in each host: as a single 2A peptide‐linked “operon” (Chng et al., 2015), as convergent split cassettes at the same locus, or as cassettes at different loci. Various other secretion tags, like those from *S. cerevisiae* pre‐α factor or invertase, *P. pastoris* Kar2, or *K. marxianus* inulin were also tested for their ability to direct antibody secretion in yeasts. In filamentous fungi, a variety of HC and LC configurations including 2A linked and fusion protein designs were investigated to optimize antibody production. In *L. tarentolae*, *Leishmania mexicana* secreted acid phosphatase 2 (Sap2) tag was used to facilitate antibody secretion (Wiese, Ilg, Lottspeich, & Overath, 1995).

### 2.3 DNA assembly and transformations

Multicomponent DNA constructs were generated using DNA assembly methods as previously described (Kok et al., 2014; Serber, Lowe, Ubersax, & Chandran, 2012) and transformed into each host using methods described below.

#### 2.3.1 P. pastoris

Linear fragments of donor DNA cassettes containing ~1.0 kb of upstream and downstream homology of targeting loci to *P. pastoris* genome, guide RNA (gRNA), and vector containing *P. pastoris* ARS1 sequence and homology regions with gRNA were transformed into *P. pastoris* host strains expressing Cas9 (Cregg, Barringer, Hessler, & Madden, 1985; Horwitz et al., 2015; Weninger, Hatzl, Schmid, Vogl, & Glieder, 2016). The transformation protocol was adapted from Higgins and Cregg's electroporation method (Higgins & Cregg, 1998).

#### 2.3.2 H. polymorpha

Since there are no known episomal plasmids described for *Hansenula*, we adapted an alternative method (Horwitz et al., 2015), utilizing linear gRNA cassettes to transiently express the gRNA. *Hansenula* strains containing Cas9 were cotransformed with linear gRNA cassette(s) and linear donor DNA containing a dominant drug marker and 1.0 kb of upstream and downstream homology to the targeted loci in the *Hansenula* genome. The transformation protocol was adapted from (Faber, Haima, Harder, Veenuis, & Ab, 1994).

#### 2.3.3 A. adeninivorans

Similar to *Hansenula*, there are no known episomal plasmids described for this host. The Alt‐R™ RNA oligos system from IDT has been used in *Arxula*. The in vitro annealed RNA oligos from IDT and linear donor DNA containing ~1.0 kb of upstream and downstream homology of targeting loci to *Arxula* genome marked with an auxotrophic or dominant drug marker were cotransformed into *Arxula* with integrated Cas9 at the ribosomal DNA (rDNA) loci (Rösel & Kunze, 1996). The transformation protocol was adapted from Higgins and Cregg's electroporation method for *P. pastoris* (Higgins & Cregg, 1998).

#### 2.3.4 K. marxianus

*K. marxianus* transformation was performed using optimized *S. cerevisiae* LiAc methods (Daniel Gietz & Woods, 2002; Horwitz et al., 2015) with slight modifications in which each gRNA was gap‐repaired into an expression vector where the *K. marxianus* chromosome V CEN.ARS element replaced *S. cerevisiae* 2μ region (Orr‐Weaver & Szostak, 1983).

#### 2.3.5 *T. reesei* and *A. oryzae*

Transformation of *T. reesei* and *A. oryzae* protoplasts was adapted from a previously published protocol (Penttilä, Nevalainen, Rättö, Salminen, & Knowles, 1987).

#### 2.3.6 P. tricornutum

Transformations were attempted in *Phaeodactylum* using protocols published previously (Niu et al., 2012; Zhang & Hu, 2014). Unfortunately, these electroporation methods were unsuccessful in producing transformants on selective media in our studies, and thus antibody expression was not evaluated in this host.

#### 2.3.7 L. tarentolae

*Leishmania* was transformed with an integration plasmid containing antibody expression cassette following the manual published on Jena Bioscience's website (https://www.jenabioscience.com/images/ae3a4f50f1/EGE-1410.pdf). Antibody expression was under the control of T7 promoter fused with the TET operator.

Information on media and strain cultivation, Protein A purification, and product analysis is available in the Supporting Information Materials.

## 3 ROBUSTNESS IN INDUSTRIALLY RELEVANT MEDIA

The ability to culture each host in an industrially relevant medium amenable to scale‐up and therapeutic protein production was evaluated, except for filamentous fungi due to its long history of industrial fermentation (Cherry & Fidantsef, 2003; Kitamoto, 2002). The characteristics of desired media for industrial production include (a) a composition of readily available, inexpensive, defined chemicals (chemically defined) when feasible, (b) complex mixtures sourced only from plant or microbial sources, with no animal‐derived feedstocks (animal‐free), and (c) supportive of growth under dark conditions, since light requirements may limit fermenter scale‐up (heterotrophic vs. photosynthetic or mixotrophic).

In summary, the yeasts all grew well in animal‐free heterotrophic media as expected (Figure S1). Comparing carbon sources, *A. adeninivorans* grew well‐using sucrose, a relatively inexpensive carbon source compared with glycerol. The most affordable carbon source, lactose, did not support growth in *H. polymorpha* and *P. pastoris* and only limited growth in *K. marxianus*. Industrial‐friendly heterotrophic conditions could not be found for the diatom (Table S1). Animal‐free media were identified for *L. tarentolae*, but the growth rate was reduced (Table S3). Further work will be required before this host system would be suitable for large‐scale production of therapeutic proteins. Further details on the culture studies are available in the Supporting Information Materials.

## 4 TRACTABILTY OF GENOME ENGINEERING

The molecular toolkit available for genetic alterations has substantially advanced over the past decade. These novel genetic tools provide the ability to quickly modify organisms with efficiency and are highly functional in most microbial systems (Horwitz et al., 2015; Wagner & Alper, 2016). For a new host, a key question is what would it take in terms of time, resources, and effort for a group to get these tools operational and running? These factors are relevant for any company seeking to complement a CHO‐based platform. Table 2 outlines some of the key characteristics of these hosts that are important in the efficiency and speed with which they could be engineered to express therapeutic proteins.

**Table 2:** Key characteristics of selected hosts important in genetic engineering.

Host	Doubling time (hr)	Efficiency of single change (%)	Multiplex changes (max number/efficiency)	Cycling time in weeks (confirmed change is made)	Number of genes changed per month	Time for HC/LC integration to bank made (months)^a^
*Pichia pastoris*	2	>90	5 (30%)	2	10	1–2
*Kluyveromyces marxianus*	1.2	8–92	2 (12%)	2	4	1–2
*Hansenula polymorpha*	2	>80	3 (10–60%)	2	6	1–2
*Arxula adeninivorans*	2	50–100	3 (17%)	2	6	1–2
*Trichoderma reesei*	3.5	1–30^b^	NA	10	0.4	4
*Aspergillus oryzae*	2.5	1–30^b^	NA	10	0.4	4
*Cricetulus griseus (CHO)*	19–25	<1^c^	2 (<1%)	14–16	0.5	6

*Note*. *L. tarentolae* was not included, since this was plasmid‐based transformation; while no expression could be achieved for *P. tricornutum.*

^a^ Time to introduce and confirm change plus expansion of confirmed culture to banking.

^b^ Without NHEJ deletion.

^c^ The efficiency will depend on transfection method and selection system.

Electroporation techniques have previously been reported for diatoms (Niu et al., 2012; Zhang & Hu, 2014). We were not successful in introducing DNA into *P. tricornutum* using these methods. Microprojectile bombardment has also been used to test a variety of selectable markers and reporter genes, but this technique is out of reach for most laboratories (Niu et al., 2012). We did not pursue this host further here.

We tested the efficiency with which targeted and marker‐less single, double, triple, quadruple, and quintuple integrations of genes can be made using CRISPR in *P. pastoris*. We modified a reported method for editing *P. pastoris* with CRISPR/Cas9 (Weninger et al., 2016). Cas9 was integrated into *P. pastoris* and expressed under a native promoter *pPGK1* with medium expression. These cells were transformed with a linear Nat‐marked vector backbone, gRNAs under a constitutive promoter and donor DNAs. The gRNA cassette contains RNA polymerase II promoter *pHTA1* from *P. pastoris* (Weninger et al., 2016), 19mer gRNA sequence, structural gRNA sequence, and *ADH1* terminator from *Saccharomyces*. The gRNA was flanked by hammerhead (HH) and hepatitis delta virus (HDV) ribozyme sequences (Weninger et al., 2016). This modified system for editing with CRISPR/Cas9 in *P. pastoris* was highly efficient for targeted integration with up to 100% efficiency for single, double, triple, up to 47% for quadruple, and 30% efficiency for quintuple integrations (Table 2).

We also developed an approach for CRISPR‐based engineering in *K. marxianus*. Initial testing using a CRISPR system developed for *Kluyveromyces lactis* (Horwitz et al., 2015) resulted in low efficiencies (10% success rate for a single genomic integration). Subsequent experiments demonstrated that development of a stable plasmid (Iborra & Ball, 1994) for the expression of the gRNA component was crucial and efficiency reached up to 92% for a single integration (Table 2). Adapting the multiplex engineering method originally developed for *S. cerevisiae* to *K. marxianus* (Horwitz et al., 2015) resulted in simultaneous double integrations with 12% success (Table 2).

Targeted single, double, and triple integrations in *H. polymorpha* were also successful using CRISPR (Table 2). These results were achieved by transforming *H. polymorpha* cells, containing an integrated Cas9 under the native constitutive promoter *pFBA1*, with linear gRNAs and donor DNAs. The linear gRNA cassette contains the RNA polymerase III promoter *pSNR52* from *Saccharomyces*, 19mer gRNA sequence, structural gRNA sequence, and *SUP4* terminator from *Saccharomyces*. This new *Hansenula* CRISPR system is highly efficient for targeted integration with up to 100% efficiency for single integrations, up to 45% for double, and up to 64% for triple integrations.

Similar to all other three yeasts, a high‐efficiency CRISPR tool was successfully developed in *A. adeninivorans* targeting single, double, and triple loci (Table 2). *A. adeninivorans* cells, containing an integrated Cas9 under the native constitutive promoter *pTDH3*, were transformed with in vitro annealed RNA oligos and donor DNAs. This new *Arxula* CRISPR system is highly efficient for targeted integration with up to 100% efficiency for single and double integrations, and up to 17% for triple integrations.

The success of implementing CRISPR‐Cas9 in these hosts for genome editing greatly simplify the process of strain engineering. For example, the time required to make 20 individual changes in the genome of a strain could be reduced from 40 weeks to as little as 8–12 weeks.

Expression in the filamentous fungi has traditionally used random integration, though CRISPR‐Cas9 techniques have been developed (Katayama et al., 2015; Liu, Chen, Jiang, Zhou, & Zou, 2015). Due to long strain engineering cycle time required for *T. reesei* and *A. oryzae*, and because our priority was to produce full‐length antibody, we utilized more established random integration techniques to examine antibody expression in these two filamentous hosts. There has been no reported work using CRISPR‐Cas9 in *L. tarentolae* for gene editing, thus we relied upon the commercially available protein expression kit for antibody expression.

## 5 ANTIBODY PRODUCTION IN ALTERNATIVE HOSTS

### 5.1 Production of an immunoglobulin G (IgG) in yeasts

**Figure 1:**
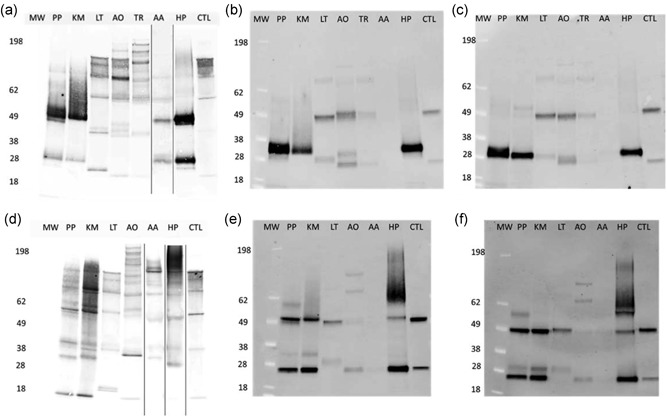
Western blot analysis for anti‐CD20 (a–c) and Herceptin (d–f) produced from seven microbial hosts. (a and d) Nonreduced gels on ProA purified samples. (b and e) Reduced gels on ProA purified samples. (c and f) Reduced gels for ProA purified and Endo H‐treated samples. Please note that different dilutions were made to these samples based on final concentrations of the antibodies purified from these hosts. AA, *Arxula adeninivorans*, Y412aa and Y858; AO, *Aspergillus oryzae*, Y960 and Y976; CTL, antibody produced from CHO cells; HP, *Hansenula polymorpha*, Y138 and Y023; KM, *Kluyveromyces marxianus*, Y350 and Y486km; LT, *Leishmania tarentolae*, Y396 and Y935; MW, molecular weight ladder; PP, *Pichia pastoris*, Y242 and Y324; TR, *Trichoderma reesei*, Y385

We first sought to express an IgG1 antibody developed with specificity to CD20. Attempts to express this anti‐CD20 antibody were not successful in all four yeast hosts. Although all four hosts produced a comparable amount of antibodies at approximately 1–10 μg/ml range on Octet (Table S2), no full‐length antibody was observed by western blot analysis. All four yeast hosts secreted similar ~60 kDa fragments that reduced to ~30 kDa, perhaps implicating a common limitation in expression or degradation (Figure 1). This lack of full‐length anti‐CD20 antibody was unexpected since other full‐length IgG constructs have been successfully expressed in minimally engineered strains of *P. pastoris*, including Herceptin and Rituxan (Li et al., 2006; Shah et al., 2015; Zha, 2013).

We sought to determine whether protease activity, either secreted or intracellular, was responsible. Incubation of anti‐CD20 antibody with production media from wild‐type strains after removal of the cells (“spent media”) did not result in degradation of the antibody, even after 23 hr (Figure S2). In contrast, incubation of the anti‐CD20 antibody with cell lysates from each of the four species led to rapid degradation of the antibody (Figure S2). Heat inactivation of the cellular lysates prevented degradation (but induced aggregation). These data together support the enzymatic proteolysis of the protein. They also suggest secreted proteases were not responsible for the observed degradation in these yeast strains, and that the degradation occurs intracellularly. It is possible that the cultivation conditions of these strains may also have increased cell lysis thus releasing proteases to the media, which led to antibody degradation extracellularly. Nevertheless, it does not seem that antibody degradation resulted from proteases secreted from wild‐type yeasts.

We analyzed the ~30 kDa fragment produced by *P. pastoris* using mass spectrometry. These data revealed cleavage between two lysine residues K214–K215, consistent with the consensus motif for the protease yapsin 1 (Gagnon‐Arsenault, Tremblay, & Bourbonnais, 2006). These residues are located in the constant region of the HC and cleavage yields a ~26 kDa N‐terminal fragment of the HC. KK is a potential recognition motif for the protease yapsin, however, deleting *yps1* gene in *H. polymorpha* did not result in any secreted product. It has been reported that YPS1 can mediate the processing of the pre–pro‐α leader sequence, allowing for KEX2‐independent processing in yeast (Egel‐Mitani, Flygenring, & Hansen, 1990; Werten & de Wolf, 2005). Therefore, the deletion of *yps1* in *H. polymorpha* may have interfered with the processing of the pre–pro‐α leader, and thus inhibited secretion of the antibody or the truncated fragment. Changing KK to KT in the anti‐CD20 in *P. pastoris* or *H. polymorpha* did not change the product profile (data not shown), suggesting other unknown activity in the cells must be involved.

### 5.2 Production of full‐length IgG1 in yeasts

Since the expression of the anti‐CD20 antibody sequence was inconsistent with prior experiences producing antibodies in some of these yeasts, we also tested expression constructs for Herceptin and Rituxan. Constructs were designed as convergent, split expression cassettes using a shorter pre‐α leader in place of the pre–pro‐α leader previously used. We chose this shorter leader to match constructs used in successful published efforts (Zha, 2013), though the longer leader has also been used successfully as well (Kozlov & Yagudin, 2008). We also tested the same secretion tag from Kar2 for Rituxan, as published by Glycofi in *P. pastoris* (Li et al., 2006). Using these new constructs, we detected the expression of full‐length IgG in all four yeast strains (Figure 1).

#### 5.2.1 P. pastoris

Protease‐deleted (*pep4Δ*) *P. pastoris* strains expressed both Herceptin and Rituxan in full‐length (Figure 1 and Figure S3). Under nonreducing conditions, a diffuse 150 kDa + band matching the CHO reference was observed for both constructs. In addition, a potential kexin‐specific protease site in the LC construct was mutated (“KR to TR”) to test whether or not degradation of LC may influence the titer observed. We did not find extensive cleavage of LC in *P. pastoris* (Figure S3). The remaining smeared protein bands above the HC from Endo H‐treated samples (Figure 1f, lane PP) suggests possible O‐linked mannosylation of the HC (Nett et al., 2013).

Several genes involved in oxidative protein folding in ER and essential for maintaining ER redox balance *ERO1* (Gasser, Sauer, Maurer, Stadlmayr, & Mattanovich, 2007; Harding et al., 2003), protein sorting *VTH1* (Cooper & Stevens, 1996) as well as a heat shock protein of the HSP70 family Ecm10 (Baumann, Milisav, Neupert, & Herrmann, 2000), and ER molecular chaperone Cne1 (Kimura et al., 2005; Parlati, Dominguez, Bergeron, & Thomas, 1995; Zirpel et al., 2018) have been reported as beneficial for expression of recombinant proteins in yeast. We tested how efficiently we could overexpress these genes in an engineered strain using the CRISPR/Cas9 system. Combinations of up to four of these genes under *pTDH3* were knocked into a *P. pastoris* strain producing Herceptin. The resulting strain Y676 exhibited about 40% higher titers than the parent Y324 (Table S2). This result demonstrated that engineering the host's genome using CRISPR/Cas9 can allow for rapid cycles of strain development to improve expression.

#### 5.2.2 A. adeninivorans

In *A. adeninivorans,* Herceptin and Rituxan were both fused to the shorter secretion leader sequence pre‐α from *S. cerevisiae*, and expressed as convergent, split expression cassettes. Both antibodies were expressed under weak native promoters and integrated at the *LYS2* locus in the base strain Y946. In contrast to *P. pastoris,* where full‐length antibody was detected for both Herceptin and Rituxan*,* full‐length antibody was only detected in strains expressing the Herceptin antibody cassette (Figure 1). Although initial production appears very low, we achieved full‐length antibody production in a single engineering cycle. Production could be further improved through increasing promoter strength, deletion of proteases, and overexpression of chaperones and other proteins.

#### 5.2.3 K. marxianus

In *K. marxianus*, codon‐optimized sequences of heavy and LCs of Herceptin and Rituxan were initially integrated at either *YKU80* or *PRB1* locus using constructs designed as convergent, split expression cassettes. Both Herceptin and Rituxan used the shorter pre‐α leader sequence and gene expression was driven using the native strong glycolytic promoter *pTEF*. We assessed the secretion of the full‐length antibody under two fermentation conditions (standard and cell recycle). Cell recycle allowed high cell density production under conditions of minimal growth. *K. marxianus* secreted both full‐length Herceptin and Rituxan and the process using cell recycle improved titers significantly for all the strains tested (Figures 1 and S4). Expression or secretion of Herceptin was locus dependent: secretion of one copy of Herceptin from the *YKU80* locus gave improved protein production/secretion compared with expression from *PRB1* locus (data not shown).

To determine the effect of copy number on the secretion of a monoclonal antibody, we integrated a second and a third copy of Herceptin at the *PEP4* locus and the *PEP4/PRB1* loci (integration confirmed by colony PCR at these loci), respectively. Increasing copy number improved titers significantly in a monotonic manner (Table S2 and Figure S4). The strain with three copies of Herceptin Y629 produced a titer of 17.9 ± 0.5 μg/ml (*N* = 8, as measured by Octet), which was seven‐fold higher than the grandparent strain Y487 with one copy of Herceptin. The titer was similar to the range observed for *P. pastoris* with minimal engineering. This result confirms that varying copy number can provide a useful means to improve productivity in yeast as is common in cell line development for CHO.

#### 5.2.4 H. polymorpha

Unlike *A. adeninivorans*, *K. marxianus*, and *P. pastoris*, initial attempts to express Herceptin and Rituxan in *H. polymorpha* resulted in a fragment of ~60 kDa that was also observed with the anti‐CD20 antibody (Figure S5). Whereas the sequences of the three antibodies tested seem to have a significant effect on the secretion of full‐length antibody in the other three yeast hosts, the differences do not appear in *H. polymorpha*.

We hypothesized the observed fragment was a product of degradation. It has been reported in the literature that the deletion of yapsin 1 (Yps1), an aspartyl protease, reduced the level of protein degradation activity in *H. polymorpha* (Sohn et al., 2012). Furthermore, in a different methylotrophic yeast, *Ogataea minuta*, deletion of *YPS1* alone or in combination with *PEP4* and *PRB1*, reduced the degradation of IgG HC (Kuroda et al., 2007). Therefore, we constructed two new protease‐deficient strains, Y840 (*yps1Δ*) and Y842 (*yps1Δ pep4Δ prb1Δ*), to use as new base strains with which to produce antibodies. In addition to testing different protease deletions, we also tested the effect of promoter strength and secretion tag on antibody production. Up to this point, all three antibodies were fused to either the *S. cerevisiae* pre–pro‐α or pre‐α secretion tag, and expressed under the strong methanol inducible promoter *pMOX1*, both of which are commonly used for heterologous protein expression in this host. We also observed from the initial work with the anti‐CD20 antibody, the titers of the degraded fragments (as measured by Octet) were always higher in strains where a mannosyltransferase *OCH1* was deleted (Table S2), suggesting *OCH1* deletion is beneficial for antibody secretion by reducing the degree of hyper‐glycosylation—a known challenge for expression of recombinant proteins in yeast (Matthews et al., 2017; Tang et al., 2016). Combining all these parameters, a new set of Herceptin expression constructs were designed with (a) constitutive promoter *pTDH3*, (b) new secretion tag from *S. cerevisiae* invertase Suc2, and (c) targeting the deletion of *OCH1*. The new constructs were transformed into Y578 (wild type), Y840 (*yps1Δ*), and Y842 (*yps1Δ pep4Δ prb1Δ*; Table S4).

The full‐length antibody was detected in all three base strains by western blot analysis (Figure S5). Thus, the combination of reducing the expression level of the antibody by switching from the methanol inducible promoter *pMOX1* (the strongest promoter in *H. polymorpha*) to the weaker promoter *pTDH3* (50% strength relative to *pMOX1*; Ravin et al., 2013) and the deletion of *OCH1* was important for producing full‐length antibody. These data suggest that the balance between protein expression and secretion is very important for the proper assembly and export of the antibody. The data also confirmed that Yps1 was the protease responsible since its deletion completely eliminated the ~60 kDa degradation fragment (Figure S5, lanes 2–5 vs. lanes 7–9). Interestingly, mutation of the *YPS1* cleavage site in the HC (KK to KT) did not eliminate the degradation, suggesting some degree of promiscuity in the consensus sequence recognized by this enzyme or proteolytic activity by a different enzyme (data not shown). The role Yps1 plays in the degradation of the HC of IgG is unknown. Degradation of the antibody was further reduced in the triple protease‐deficient strain, Y842 (Figure S5, lanes 11–13). The relative ease with which the genome was altered to remove protease and enzymes involved in glycosylation further highlights the accessibility of multigene editing possible in yeast and the rapid cycles of iteration in design.

Interestingly, protease susceptibility varied among the three antibodies, suggesting the modest molecular design of the antibodies could also provide another means for modulating degradation. As noted above, none of the yeast hosts secreted full‐length anti‐CD20, even when the known proteases *pep4* and *prb1* were engineered out (Table S2). All four yeasts could produce full‐length Herceptin with minimal engineering, however, indicating the variable regions of IgG antibody constructs may influence secretion in the yeasts.

### 5.3 Filamentous fungi

Filamentous fungi are used extensively to produce industrial enzymes and examples of monoclonal antibody expression have been shown in several hosts including *T. reesei* (Baldwin et al., 2006). Historically, the expression of heterologous proteins in filamentous fungi has used random integration. The impairment of NHEJ components (Krappmann, 2007) and the use of split‐markers (Chung & Lee, 2015) have increased efficiency for targeted integration, and CRISPR‐Cas9 techniques allow highly efficient targeted integration (Katayama et al., 2015; Liu et al., 2015). As mentioned previously, establishing these techniques would take dedicated time and iterations to establish suitable engineered strains as foundations for further engineering. Therefore, we relied on random integration to produce initial strains that expressed antibody.

**Figure 2:**
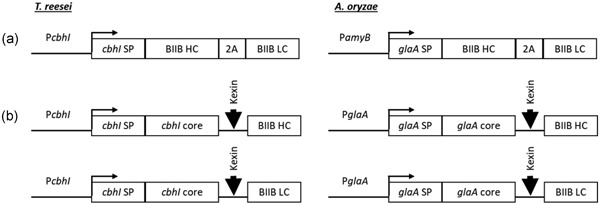
Configurations of antibody expression cassettes in *Trichoderma reesei* and *Aspergillus oryzae*. (a) HC and LC are linked by a 2A linker. (b, left) *T. reesei* CBH1 catalytic core linked to codon‐optimized HC and LC. A kexin protease cleavage site is added to the end of the coding sequence of the CBH1 linker sequence to facilitate cleavage of the fused proteins during secretion. (b, right) *A. oryzae* GlaA catalytic core linked to codon‐optimized HC and LC. A kexin protease cleavage site is added to the end of the coding sequence of the GlaA to facilitate cleavage of the fused proteins during secretion. CBHI: cellobiohydrolase 1; GlaA: glucoamylase A; HC, heavy chain; LC, light chain

The anti‐CD20 and Herceptin antibodies were expressed in *T. reesei* and *A. oryzae* using two genetic constructs*.* The first (Figure 2A) used codon‐optimized genes for HC and LC linked by a 2A linker. Neither organism expressed significant amounts of antibodies in this configuration (barely detectable by dot blot with estimated titers below 32 ng/ml; data not shown). Polycistronic gene expression is not commonly used in filamentous fungi; we speculate that either the 2A linker is not compatible with these organisms or the lack of a native carrier protein inhibited the secretion of a human antibody.

A second configuration (Figure 2b) used a carrier protein to help facilitate the secretion of the HC or LC independently (Baldwin et al., 2006; Ward, Wilson, Kodama, Rey, & Berka, 1990). Native carrier proteins can increase expression of heterologous proteins in various filamentous fungi, including the use of cellobiohydrolase 1 (CBHI) in *T. reesei* (Keränen & Penttilä, 1995) and glucoamylase A (GlaA) in *A. oryzae* (Tsuchiya, Gomi, Kitamoto, Kumagai, & Tamura, 1993; Ward et al., 2004). The HCs or LCs were fused to the CBHI catalytic core domain (*T. reesei*) or the GlaA core domain (*A. oryzae*), lacking the cellulose or starch binding domain, respectively, with a kexin protease cleavage site. The Kex2 protease is a native Golgi protease that cleaves after the dipeptide sequence KR during secretion. In all configurations, a native signal peptide from CBHI or GlaA (for *T. reesei* and *A. oryzae*, respectively) was used to direct secretion.

*T. reesei* expressed about 2 μg/ml of anti‐CD20 antibody (estimated by immunoblot of supernatants from plates or flasks). A reduced western blot analysis of the fusion transformants showed three significant bands running at 25, 50, and 80 kDa (Figure 1). These bands presumably represent the LC, HC and a fusion protein of CBH1 core domain with linker and LC that was not cut by the kexin protease during secretion. Insufficient cleavage by the kexin protease has been observed previously in *T. reesei* and *Aspergillus niger* (Ward et al., 2004). A nonreduced western blot analysis showed more than a dozen bands (Figure 1). Based on the size of these bands, we expect that they correspond to HC, LC, and full‐length anti‐CD20 antibody, as well as many additional bands corresponding to non‐kexin cleaved LC and HC fusions with CBH1 protein and additionally combined as dimers and tetramers.

*A. oryzae* produced ~1.3 µg/ml of anti‐CD20 antibody (measured by Octet). Running these samples on a western blot analysis (Figure 1) show a banding pattern similar to that from *T. reesei*. A nonreduced western blot analysis shows bands for both the full‐length HC and LC fusion as well as many bands running both smaller and larger than the anti‐CD20 standard. A reduced western blot analysis shows two significant bands representing the HC and LC as well as a ladder of minor bands.

Using the design with a fused carrier protein, *A. oryzae* did express Herceptin (Figure 2b). As part of this evaluation, we altered the linker between the units from ISKRGGG—used previously in *T. reesei* (Ward et al., 2004), to a YKR. Consistent with that prior study, cleavage of the shorter sequence was less efficient than the longer one observed in the anti‐CD20 antibody constructs (Figure 1).

Secreted proteases from the filamentous fungi reduced our ability to quantify and test antibody produced from these hosts. Filamentous fungi secrete significant amounts of proteases that can degrade the desired product (Landowski et al., 2015). The native activity of these proteins presumably impacts the observed titers seen in *T. reesei* and *A. oryzae*. To evaluate the extent of this problem, we spiked purified anti‐CD20 antibody into spent production media from the wild‐type strain of each species and monitored the full‐length antibody quality over time by western blot analysis (Figure S6). Spent media from both *A. oryzae* and *T. reesei* degraded the anti‐CD20 antibody to varying extents. In the case of *A. oryzae*, a ladder of lower molecular weight bands of increasing intensity and extent over time was observed with concomitant disappearance of the full‐length band. Degradation was observed even at the shortest time tested, indicating near instantaneous activity upon the purified protein. Degradation of the antibody was also observed in *T. reesei* spent production media, though to a lesser extent, as evidenced by the increasing appearance of a ~100 kDa band with loss of the full‐length antibody.

The addition of known protease inhibitors (phenylmethylsulfonyl fluoride [PMSF; 1 mM], Pepstatin A [1 μM], 4‐(2‐aminoethyl)benzenesulfonyl fluoride hydrochloride [AEBSF; 1 mM], and the cOmplete^TM^ Protease Inhibitor Cocktail) had minimal effect. PMSF had a minor effect, but surprisingly, AEBSF and the cOmplete^TM^ cocktails had no detectable effect. Higher concentrations may be necessary without strain engineering. For *T. reesei,* protease activity was reduced by using a piperazine‐1,4‐bis(propanesulfonic acid)(PIPPS)‐buffered media with pH control (data not shown). Knock‐outs of these proteases are another route to reducing proteolytic degradation in these hosts (Landowski et al., 2015). In summary, both filamentous fungi hosts required fusion proteins for sufficient secretion of the antibody. The efficient cleavage of fusion from the HC and LC remains an issue for these hosts, as will removal of the fused chaperone protein in downstream operations. Further engineering of the secretory path to include other chaperones may also enable reduced reliance on fusion constructs.

### 5.4 Protozoa

Using a commercially available integrating expression vector (Jena Biosciences), *L. tarentolae* secreted both full‐length anti‐CD20 and Herceptin antibody. The banding patterns on a gel were similar to the anti‐CD20 and Herceptin antibody secreted from CHO cells (Figure 1). In BHI media, anti‐CD20 antibody titer reached ~4 μg/ml estimated by Octet, comparable with the titers from the four yeast hosts expressing anti‐CD20 or Herceptin (Table 2). In our alternative YPD media with porcine hemin added, the titers were approximately half those observed in BHI medium, as estimated by western blot analysis (data not shown), primarily due to lower biomass achieved. These results suggest the baseline productivity of the protozoan is solid, but slow growth rates present a key challenge for large‐volume production since it impacts both the amounts of raw materials required and time for facility use.

## 6 MOLECULAR PROPERTIES OF EXPRESSED ANTIBODIES

**Figure 3:**
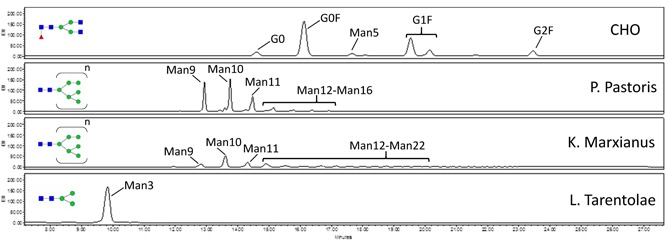
*N*‐glycan analysis of Herceptin producing in various strains—CHO (a), *Pichia pastoris* (b), *Kluyveromyces marxianus* (c), and *Leishmania tarentolae* (d). CHO, Chinese hamster ovary [Color figure can be viewed at wileyonlinelibrary.com]

Monoclonal antibodies have been previously expressed in some of these hosts, but the examples produced here allowed direct comparison of the same proteins from all tested hosts (Figure 1). The two antibodies here were diverse with respect to the degree of antibody engineering, format, and charge profiles. Several hosts produced sufficient amounts of antibodies for glycan analysis. The *N*‐glycan profiles of Herceptin from two yeasts (*P. pastoris* and *K. marxianus*) and anti‐CD20 from the protozoan (*L. tarentolae*) were analyzed by UPLC. For both *P. pastoris* and *K. marxianus*, we observed various identifiable high mannose species in the glycan profile (Figure 3). These results were expected, based on previous reports that yeasts hyper‐mannosylated secreted proteins (Wildt & Gerngross, 2005). Purified antibody from the yeast resulted in well‐defined HC bands with faster mobility in western blot analysis when treated with the glycan trimming enzyme Endo H. These results confirmed the known glycan issues of these yeasts that can be address by engineering (Beck, Cochet, & Wurch, 2010; Li et al., 2006; Zha, 2013), but importantly did not reveal other unexpected posttranslational modifications.

Interestingly, *L. tarentolae* uniformly generated Man3 glycosylation. The Man3 glycan structure is commonly observed in antibodies produced in mammalian cells but is usually found at very low levels compared with other glycan structures. Thus, little is known regarding the pharmacokinetic/pharmacodynamics properties or impact on functionality. Nonetheless, Man3 is an interesting, and potentially advantageous, starting point for further glycoengineering toward more complex glycan structures commonly observed in mammalian cell cultures.

## 7 DISCUSSION

Over the last decade, several expression systems have been reported as alternatives to mammalian cells. However, the development of these systems has been fragmented, making it difficult to compare systems for their potential advantages over CHO cells. Here, we sought to examine several hosts directly head‐to‐head. For this purpose, we started with wild type, unengineered expression systems for following two reasons: (a) to assess the potential time required for independent development of each and (b) to generate data “in‐house” to corroborate publications on particular expression systems of interest. The systems evaluated here were all as selected potential alternatives to CHO cells based on previously articulated criteria (Matthews et al., 2017).

*L. tarentolae* produced and secreted full‐length antibodies with uniform Man3 glycan structures. This host would require glycoengineering to generate more complex glycoforms, but these results are encouraging since Man3 is an acceptable starting point. The molecular properties of these antibodies were the most similar to those from mammalian cells in this study. Next steps for *L. tarentolae* would include optimization of the fully animal‐free and hemin‐free culture media to support higher cell densities and faster growth rates than that found here. The robustness of this host in animal‐free media is critical to compete with CHO.

*P. tricornutum* must be enabled for heterotrophic growth to be suitable for producing therapeutic proteins cost effectively. While not evident in our experiments, fully heterotrophic growth has been reported for *P. tricornutum* with the addition of a heterologous glucose transporter gene, *glut1* (Zaslavskaia et al., 2001). Importantly, it is essential to create improved methods to transform the host with recombinant DNA, preferably by chemical transformation. Electroporation may be feasible, but the typical protocols were not successful in this study. Bacterial conjugation has yielded successful transfection of *P. tricornutum*, but such methods are time‐consuming and inefficient (Karas et al., 2015).

The filamentous fungi presented a major challenge with respect to tractability, and thus, engineering in these species was limited to random integration of antibody cassettes here. Full‐length anti‐CD20 and/or Herceptin antibody was detected in *A. oryzae* and *T. reesei*, but titers were relatively low. Our results showed that codon optimization and antibody configurations play important roles in antibody expression. Protease degradation is clearly an issue in these strains. Titers in the fermentation of *T. reesei* expressing Herceptin can diminish from over 4 g/L at 66 hr to less than 1 g/L by 167 hr (Baldwin et al., 2006). Deletion of six protease genes can mitigate the degradation of HC antibody (Landowski et al., 2015). Deletion of major cellulase genes, which constitute more than 85% of the secreted protein in *T. reesei*, should lessen competition for transcription and secretion with the antibody fusion constructs. Improved cycles for strain engineering using tools like CRISPR/Cas9 are essential given the number of edits required to overcome these constraints and those for altering native glycosylation.

All four yeasts evaluated showed the ability to produce full‐length antibodies and were amenable to rapid, efficient genomic engineering with modern tools like CRISPR/Cas9. The productivity observed was low, though further engineering to include chaperones and foldases is likely to improve the expression of full‐length antibody. With the highly efficient multiplexing CRISPR/Cas9 tools developed in all yeast hosts in this study, the speed of strain engineering will be greatly increased to enable rapid improvement in antibody productivity. As a proof of concept (Table S2), one round of engineering introduced four genetic changes in *Pichia* (from Y324 to Y676), and improved titers of Herceptin by 40%. The significance in titer improvement was also seen in *K. marxianus* (Table S2) where two sequential changes from Y487 to Y631 and Y629 improved titers of Herceptin seven‐fold. Although significant improvements in productivity will be needed to compete with the levels currently seen in CHO cells, future directions to increase antibody productivities and qualities, include but not limited to rational strain engineering, directed evolution, mutagenesis, and so forth have a high likelihood of leading to highly productive strains.

As expected, the antibodies exhibited highly mannosylated HCs, but engineering pathways to generate complex, mammalian‐like glycan structures have been demonstrated. (Beck et al., 2010; Bobrowicz et al., 2004; Choi et al., 2003; Hamilton et al., 2006; Wildt & Gerngross, 2005). The highly efficient, multiplexed, and marker‐less genome editing facilitated by CRISPR/Cas9, however, provide a substantial advantage for achieving such engineering in significantly less time than previously required for such improvements. An unexpected finding in these studies was the influence of the antibody sequence on their expression in yeasts. The anti‐CD20 and Herceptin antibodies had highly similar amino acid sequences: 90% for HC and 92% for LC. Nonetheless, the profiles of the secreted products were varied among the four yeasts. None produced full‐length anti‐CD20, while Herceptin was successfully expressed in all. The HCs for both antibodies contain a potential cleavage motif for yapsin (KK) and the LCs contain one for kexin (KR). Attempts to mitigate the sequence in the HC at the cleavage site were not successful here; the variable regions may also impact the differences observed between these two antibodies. These results suggest a further understanding of secretory requirements in yeast is needed to achieve broad utility for many constructs.

This study and others demonstrate the potential to look beyond CHO for low cost, high productivity systems for therapeutic protein production. Unlike CHO cells, there has not been a sustained investment to date in alternative expression systems to determine how they might provide suitable alternatives for producing recombinant biopharmaceuticals. Increasing needs to support patient populations orders of magnitude greater than the current one, to reach emerging markets at low cost, and to meet acute epidemics quickly are all incentives for industry and academic partners to develop complementary alternatives to the CHO‐based platforms that are now widely established. For adoption of an alternative host at this time, the time and resources needed to engineer an operational host (remove proteases, convert glycosylation patterns, develop easily interchangeable expression systems) must be a key consideration for any company interested in developing such a system. Speed in cell line development for individual products is also an important factor. Developing commercial‐grade cell lines depends not only on the growth rate of the cells but also the ease and speed at which several changes can be introduced to maximize the production levels. In this study, we have demonstrated this growth and cycling time advantage with yeasts. Overall, the study here has established a basic framework for evaluating the suitability of an alternative expression system that is relevant for industrial‐scale production of biopharmaceuticals in comparison with the currently preferred host, CHO cells. All the hosts require additional development, but the tractability of genome editing tools and defined characterization of the products expressed here provide a foundation for future advancement of one or more of these hosts that could offer speed, quality, and volumetric productivities to enable new markets and access for protein biopharmaceuticals. Lessons learned in developing alternative hosts could also inform further engineering of current standards like CHO cells.

## Supporting information

Supplementary information
