# Supplementary material for: Challenging the workhorse: Comparative analysis of eukaryotic micro‐organisms for expressing monoclonal antibodies
Source: Biotechnol Bioeng. 2019 Mar 7;116(6):1449–62. doi: 10.1002/bit.26951 (PMC6836876; doi:10.1002/bit.26951)

**Challenging the workhorse: Comparative analysis of eukaryotic microorganisms for expressing monoclonal antibodies**

Hanxiao Jiang^a, 1, 2^, Andrew A. Horwitz^a, 1^, Chapman Wright^b^, Anna Tai^a^, Elizabeth A. Znameroski^a^, Yoseph Tsegaye^a^, Hailley Warbington^a^, Benjamin S. Bower^a^, Christina Alves^b^, Carl Co^b^, Kanvasri Jonnalagadda^b^, Darren Platt^a^, Jessica M. Walter^a^, Venkatesh Natarajan^b^, Jeffrey A. Ubersax^a^, Joel R. Cherry^a^, and J. Christopher Love^b^

Affiliations

^a^ Amyris Inc., 5885 Hollis Street, Suite 100, Emeryville, California 94608, USA.

^b^ Biogen, 225 Binney St, Cambridge, Massachusetts 02142, USA

^1^ H.J. and A.H. contributed equally to this work.

^2^ To whom correspondence should be addressed. E-mail: jiang@amyris.com

**Supplemental Text, Figures and Tables**

Methods and Materials

**Media and strain cultivation**. Production of antibodies from four yeast hosts was conducted in cultures in 96-well microtiter plates (1.1 or 2.2 mL) at 1,000 rpm shaking with 80% relative humidity in the media described below. Cells were typically grown for 1-2 days (pre-culture phase) before being diluted or spun down and resuspended in fresh media and re-grown for 2-3 days (production phase). Cells were separated by centrifugation and supernatant samples were collected for future analyses. To produce large volume of cultures, cells were grown in 50 mL of media in 250 mL of flasks and shaken at 200 rpm. Culturing conditions for each species are listed below.

*P. pastoris.* *P. pastoris* colonies (NRRL Y-48124) were inoculated in 360 μL of Buffered Glycerol-complex Medium (BMGY) (2% Bacto peptone, 1% Bacto yeast extract, 100 mM potassium phosphate pH 6.0, 1.34% yeast nitrogen base without amino acids, 0.4 µg/mL Biotin, and 1% Glycerol) and grown in 1.1 mL 96-well plates for 24 hours at 30°C. Cells were then spun down and resuspended in 360 μL Buffered Methanol-complex medium (BMMY) (2% Bacto peptone, 1% Bacto yeast extract, 100 mM potassium phosphate, pH 6.0, 1.34% yeast nitrogen base without amino acids, 0.4 µg/mL Biotin, 0.5% v/v methanol) and grown at 30°C for 72 hours. 1% v/v methanol was added to production plates after 24 and 48 hours in BMMY.

*H. polymorpha*. *H. polymorpha* colonies (NRRL Y-7560) were inoculated in 360 μL of Yeast Peptone (YP) media (2% of Bacto peptone and 1% of Bacto yeast extract) with 2% glycerol and grown in 1.1 mL 96-well plates for 24 hours at 30°C. Cells were then spun down and resuspended in 360 μL of YP media with 0.5% glycerol and 2% methanol and grown for 72 hours at 30°C.

*A. adeninivorans*. *A. adeninivorans* (NRRL Y-17692) colonies were inoculated in 360 μL of YP media with 2% glucose or glycerol and grown in 1.1 mL 96-well plates for 24 hours at 30°C. Cells were then diluted by 25-fold into YP with 4% glucose or 2% glycerol and grown for 48 hours at 30°C.

*K. marxianus*. *K. marxianus* single colonies (NRRL Y-7571) were inoculated in 1 mL of YP media with 2-4% glucose and grown in 2.2 mL 96-well microtiter plates for 48 hours at 30°C. Then, in a standard process, each culture was diluted 100-fold into fresh YP media with 4% glucose or 4% sucrose from cane syrup as sugar source and grown for 72 hours. In a “cell recycle” process, after 48 hours of pre-culture, cells were spun down and resuspended in same media as in the standard process and grown for 72 hours at 30°C.

*T. reesei.* Transformants of *T. reesei* (NRRL 15709) were screened in two sequential 2.2 mL square 96 deep-well plates. *T. reesei* spores were transferred into 600 μL of Potato Dextrose Broth and grown to mycelia at 30°C with 800 rpm shaking and 80% relative humidity for 48 hours. 100 μL of mycelia was transferred into 500 μL of 2.5% Lactose Expression Media and grown at 28°C with 1,000 rpm shaking and 80% relative humidity for 120 hours. Supernatant was obtained by filtering broth through 96-well filter plates into a 1.1 mL receiving plate.

*A. oryzae.* A small amount of *A. oryzae* spores from the transformants (NRRL 694) were transferred into a 2.2 mL square 96 deep-well plate containing 1 mL MDU2BP (Moroz, et al., 2015) and grown at 30°C with shaking at 1,000 rpm for 4 days. The supernatants were harvested by moving the mycelia mat aside and transferring the supernatant to a new plate for future analyses. Spores from a 60 mm x 15 mm plate were resuspended in 5 mL 0.01% Tween-20 and inoculated in 50 mL of MDU2BP (Moroz, et al., 2015) in 250 mL plastic baffled flasks. Shake flasks were grown at 30 °C with shaking at 120 rpm for 4 days. The supernatants were harvested by filtering through a sterile 50 mL 0.2 µm filter apparatus.

*P. tricornutum*. Culturing media and conditions were optimized in this study to enable growth of *P. tricornutum* (UTEX 646) in mixotrophic (photosynthetic + heterotrophic) conditions. An optimal medium was determined in this work and the detailed compositions evaluated are presented in the Results.

*L. tarentolae*. *L. tarentolae* colonies (Jena Biosciences) were picked in 2.2 mL 96-well plates and suspended in 1.0 mL fresh brain heart infusion (BHI), Yeast Extract (YE - 2.4% Bacto yeast extract, 1% glucose or 1% fructose, 90 mM phosphate buffer, pH 7.5), or Yeast Peptone Dextrose (YPD – YP + 2% glucose) containing 5 μg/mL hemin, 100 μg/mL zeocin, 100 μg/mL Hygromycin B, 100 μg/mL nourseothricin, and 0.5% v/v 200x Pen-Strep stock solution (<https://www.jenabioscience.com/images/ae3a4f50f1/EGE-1410.pdf>)*.*  Catalase purified from *Aspergillus niger* (Sigma cat # C3515) and peroxidase purified from horseradish (Sigma cat # 77332) were supplied to *L. tarentolae* cultures in some experiments. Cultures were grown for 72 hours at 26°C with occasional agitation before being spun down (2,000 *g* for 5 min) and re-suspended in 1.0 mL of the same media as above with 100 µg/mL tetracycline as inducer and grown at 26°C for an additional 48 hours.  Cultures were centrifuged at 2,000 *g* for 5 min and 500 µL of clarified supernatants were filtered through a nitrocellulose membrane for future analyses. Top antibody producers were run in 250 mL shake flasks containing 50 mL media and shaken at 100 rpm to achieve large volumes for *N*-glycan analysis.

**Protein A purification.** Supernatant samples from antibody production cultures were purified and concentrated using Protein A tip columns following the manufacturer’s protocol (PhyNexus, San Jose, CA, USA).

**Semi-quantitative antibody titer measurement by Dot Blot.** Dot blot analysis was carried out using the Minifold I 96-Well System (GE Healthcare, Little Chalfont, UK) according to the manufacturer protocol. Supernatants were collected from cultures grown under production conditions. Detection was performed using IRDye® 800CW goat anti-human IgG (H + L) antibody (LI-COR, Lincoln, NE, USA) as both the primary and secondary antibody and imaged on the Odyssey Infrared Imaging System (LI-COR, Lincoln, NE, USA).

**Protein sequencing**. Antibody fragments were sent to University of California at Davis (Davis, CA, USA) for sequencing analysis. N-terminal Edman determination of 10 amino acids was performed.

**Endo H treatment.** Endoglycosidases treatment was performed using Endo H_f_ (New England Biolabs, Cat. No. P0703S) according to the manufacturer’s instructions. For non-reducing samples, Glycoprotein Denaturing Buffer was replaced with 5% SDS solution.

**Western blot.** All monoclonal antibody samples were mixed with NuPAGE LDS Sample Buffer (Thermofisher, Cat. No. NP008) and denatured at 70°C for 10 min before running non-reduced samples on 3-8% Tris-Acetate precast protein gels (Thermofisher, Cat. No. EA0375). For reduced samples, NuPAGE Sample reducing Buffer (Thermofisher, Cat. No. NP009) was used as reducing agent. Reduced samples were denatured at 70°C for 10 minutes and then run on 4-12% Bis-Tris precast protein gels (Thermofisher, Cat. No. NP0321). For investigation anti-CD20 antibody degradation by intracellular cell lysate, samples were run on a 48-well E-PAGE gel using the iBlot system (Thermofisher). For Western blot analysis, Goat anti-human IgG (H + L) (LiCor, Cat. No. 925-32232) was used at a 1:10,000 dilution to detect heavy chain, light chain or full-length antibody.

**Antibody titer assay by Octet.** Purified protein for the standards were obtained from in-house programs. Titer analysis using Protein-A sensors was performed with Octet QK384 system from Pall ForteBio (Menlo Park, USA). The Octet titer assay is based on the binding rate of the protein of interest to the tip of the Protein-A sensor (Pall ForteBio, Cat. No. 18-5010). The assay consists of three steps: hydration, detection and data analysis. In the hydration step, 100 µL of culture medium was transferred into 96-well plates (Grenier Bio-One, Monroe, NC). The Protein-A sensor tips were pre-wetted for 15 min by placing the Protein-A sensor tip tray on the hydration plate. In the detection step, 100 µL of filtered culture supernatant were added to a 96-well plate and the samples were analyzed at 1200 rpm for 120 sec. Purified proteins diluted in cell culture medium were used to generate standard curve ranging from 0-100 µg/mL. In the data analysis step, binding rates were calculated using the Octet data analysis software. The protein concentrations of the samples were calculated based on their binding rates from the standard curve.

***N*-glycan analysis**. *N*-glycan analysis was performed to determine the relative abundance of *N*-linked glycan types found on monoclonal antibodies using the GlykoPrep glycan sample preparation kit (Prozyme, Cat. No. GPPNG-AB) and UPLC-HILIC (Ultra Performance Liquid Chromatography-Hydrophilic Interaction Chromatography) system. The method involved several steps. Purified protein was denatured, and immobilization of the protein was performed on separation cartridges. Deglycosylation of the proteins was performed using *N*-glycanase enzyme to release the *N*-linked glycans. The released glycans were then recovered from the cartridges and 2-Aminobenzamide labeling of glycans was performed. The excess free dye was removed using the cleanup cartridges. The detection and analysis were performed using the UPLC-HILIC system with a Fluorescence Detector.

Robustness in IndustrIALLY RELEVANT Media

The ability to culture each host in an industrially-relevant medium amenable to scale-up and therapeutic protein production was evaluated, except for filamentous fungi due to a long history of industrial fermentation (Kitamoto, 2002; Cherry & Fidantsef, 2003). The characteristics of desired media for industrial production include i) a composition of readily available, inexpensive, defined chemicals (chemically defined) when feasible, ii) complex mixtures sourced only from plant or microbial sources, with no animal-derived feedstocks (animal-free) if necessary, and iii) supportive of growth under dark conditions, since light requirements may limit fermenter scale-up (heterotrophic vs. photosynthetic or mixotrophic).

- - **Yeasts**

Animal-free media are already available for yeasts and filamentous fungi, thus we chose to evaluate the ability of each of the four proposed yeast hosts to grow in YP medium, supplemented with one of five low-cost carbon sources. Based on current pricing (Sigma-Aldrich, cell culture grade 5 or 10 kg pack sizes), the five sources from lowest to highest cost are lactose = glucose < sucrose < fructose < glycerol (SigmaAldrich, 2018). Each carbon source was tested at a 4% concentration in yeast peptone media.

After pre-growth in YPD, each host was diluted 1:40 in the test media and incubated at 25°C, 30°C, 37°C, or 44°C. Glycerol supported the highest growth for 2 of the 4 yeasts (*H. polymorpha* and *P. pastoris*), while sucrose or fructose supported the highest growth for *A. adeninivorans* and glucose, sucrose or fructose supported equal growth levels for *K. marxianus* (Supplemental Figure 1A)*.*  Except for *K. marxianus* at 44°C, none of the yeasts grew well in lactose. The lack of invertase in *P. pastoris* accounts for its lack of growth in sucrose (Sreekrishna, Tschopp, & Fuke, 1987). The optimal temperature for growth varied across the species: *A. adeninivorans* had similar growth at all temperatures, while *P. pastoris* and *H. polymorpha* grew better at lower temperatures and the optimal growth temperature for *K. marxianus* depended on the carbon source*.*

- - **Filamentous Fungi**

The Filamentous Fungi, *T. reesei* and *A. oryzae* have a long history of use in fermentation for different products. *A*. *oryzae* has been used for over 500 years in Japan to produce fermented food products including soy sauce, miso, and sake (Kitamoto, 2002) and *T. reesei* is renowned for its ability to secrete over 100 g/L cellulases (Cherry & Fidantsef, 2003). Because these hosts have a long history and protocols for fermentation conditions are readily available, no effort is needed to develop novel protocols.

- - **Diatom**

The diatom, *P. tricornutum*, requires light for growth which makes large-scale cultivation difficult. Large stirred tank fermenters do not supply sufficient illumination in standard configurations. Although photobioreactors have been developed for plant cell cultures (Huang, et al., 2017), these are difficult to scale and clean. Thus, we attempted to determine conditions for heterotrophic growth of *P. tricornutum.* Initial experiments under fully photosynthetic (~150 μmol m^-2^s^-1^ constant illumination) and mixotrophic (photosynthetic + heterotrophic with the addition of glycerol spikes every 3-5 days) demonstrated the best growth in Canadian Phycological Culture Centre (CPCC) synthetic seawater medium (Guillard & Ryther, 2018) (Supplemental Table 1). Heterotrophic growth was assessed in CPCC medium supplemented with exogenous carbon and nitrogen sources (1% glucose, 1% glycerol, 1% ammonium chloride, 1% glucose + 1% ammonium chloride, or 1% glycerol + 1% ammonium chloride), all in the absence of light, but no growth was evident after nine days. This extended lag exceeded the basal lag phase by 3-fold, indicating *P. tricornutum’s* inability to grow in solely heterotrophic conditions due to its inability to take up nutrients during the dark cycle (Terry, Hirata, & Laws, 1983). A 1:23 light:dark cycle increased growth slightly, especially in the presence of glycerol, while a 12:12 light cycle demonstrated steady growth over 10 days (Supplemental Figure 1B and Supplemental Table 1). These data indicate that some illumination is necessary for heterotrophic growth of *P. tricornutum* without extensive engineering to enable light-deficient intake of nutrients.

- - **Protozoa**

*L. tarentolae* is typically grown in Brain Heart Infusion (BHI) media and requires hemin for growth, another animal product. Thus, alternative animal-free sources of hemin were explored. Catalase purified from *Aspergillus niger* and peroxidase purified from horseradish were supplied to *L. tarentolae* cultures in various concentrations to test their effectiveness as non-animal substitutions for hemin in yeast extract (YE) media, with the hypothesis that *L. tarentolae* can sequester the non-covalently bound heme groups from these enzymes (Gaughan & Krassner, 1971). *L. tarentolae* cultures were grown in YE media (1% glucose) supplemented with porcine hemin (0.25% w/v solution in 50% ethanolamine) to a final concentration of 5 µg/mL.  Cultures were diluted (1:10) into buffered yeast extract media with varying concentrations of purified catalase or peroxidase from non-animal sources, and growth was monitored over a 94-hour period.  The growth was supported for about 70 hours in this animal product free and hemin free culture medium, with catalase supporting faster growth rates and higher cell density than peroxidase (Supplemental Figure 1C).

In a subsequent experiment, growth of *L. tarentolae* in shake plates was compared in BHI with hemin, YE media (1% glucose or 1% fructose) with hemin, and YE media (1% glucose or 1% fructose) with 500 μg/mL catalase. At 48 hours, 0.3 μg/mL of hemin or 30 μg/mL of catalase was spiked in the cultures and growth was followed up to 96 hours. *L. tarentolae* was able to achieve the same growth rates in YE media with hemin or additional hemin spike as in the BHI media with hemin or hemin spike. Although lower, growth was also achieved in animal-free medium of YE + 1% glucose + catalase (Supplemental Table 3).

In summary, the yeasts and filamentous fungi all grew well in animal-free heterotrophic media as expected. Comparing carbon sources, *A. adeninivorans* grew well using sucrose, a relatively inexpensive carbon source compared to glycerol. The most affordable carbon source, lactose, did not support growth in *H. polymorpha* and *P. pastoris* and only limited growth in *K. marxianus*. Industrial-friendly heterotrophic conditions could not be found for the diatom. Animal-free media were identified for *L. tarentolae*, but growth rate was reduced, and further work will be required before this host system would be suitable for large scale production of therapeutic proteins.

# References

Cherry, J. R., & Fidantsef, A. L. (2003). Directed evolution of industrial enzymes: an update. *Current Opinion in Biotechnology, 14*, 438-43. doi:10.1016/S0958-1669(03)00099-5

Gaughan, P. L., & Krassner, S. M. (1971). Hemin deprivation in culture stages of the hemoflagellate, *Leishmania tarentolae*. *Comparative Biochemistry and Physiology Part B: Comparative Biochemistry, 39*, 5-18. doi:10.1016/0305-0491(71)90247-1

Guillard, R., & Ryther, J. (2018, August 1). *F/2 medium using filtered seawater or artificial seawater*. Retrieved from Canadian Phycological Culture Centre: https://uwaterloo.ca/canadian-phycological-culture-centre/cultures/culture-media/f2

Huang, Q., Jiang, F., Wang, L., & Yang, C. (2017). Design of photobioreactors for mass cultivation of photosynthetic organisms. *Engineering, 3*, 381-329. doi:10.1016/J.ENG.2017.03.020

Kitamoto, K. (2002). Molecular biology of the koji molds. *Advances in Applied Microbiology, 51*, 129-153. doi:10.1016/S0065-2164(02)51004-2

Moroz, O. V., Marnta, M., Shaghasi, T., Harris, P. V., Wilson, K. S., & Davies, G. J. (2015). The three-dimensionsal structure of the cellobiohydrolase Cel7A from *Aspergillus fumigatus* at 1.5 Å resolution. *Acta Crystallographica Section F Structural Biology Communications, 71*, 114-120. doi:10.1107/S2053230X14027307

SigmaAldrich. (2018, February 22). Retrieved from Sigma-Aldrich: https://www.sigmaaldrich.com/technical-service-home/product-catalog.html

Sreekrishna, K., Tschopp, J., & Fuke, M. (1987). Invertase gene (*SUC2*) of *Saccharomyces cerevisiae* as a dominant marker for transformation of *Pichia pastoris*. *Gene, 59*, 115-25. doi:10.1016/0378-1119(87)90272-1

Terry, K., Hirata, J., & Laws, E. (1983). Light-limited growth of two strains of the marine diatom *Phaeodactylum tricornutum* Bohlin: Chemical composition, carbon partitioning and the diel periodicity of physiological processes. *Journal of Experimental Marine Biology and Ecology, 68*, 209-227. doi:10.1016/0022-0981(83)90054-0

**LIST OF SUPPLEMENTAL TABLES**

**Supplemental Table 1:**  Maximum growth rates and peak cell densities achieved in *P. tricornutum* cultures in mixotrophic conditions with varying illumination. CSH = Cold Spring Harbor synthetic seawater recipe. CPCC = Canadian Phycological Culture Centre synthetic seawater recipe. NA, not applicable.

| **Media**  **Base** | **Additives** | **Light condition**  **(light:dark; h)** | **μ_max_ (d^-1^, Log Phase)** | **Peak Cell Density**  **(cells per ml)** | |  |
| --- | --- | --- | --- | --- | --- | --- |
| CPCC | Glycerol | 24:0 | 0.304 | 11.0 x 10^6^ | at 20 d |  |
| CSH | Glycerol | 24:0 | 0.240 | 8.3 x 10^6^ | at 20 d |  |
| CPCC |  | 24:0 | 0.322 | 8.0 x 10^6^ | at 20 d |  |
| CSH |  | 24:0 | 0.231 | 5.4 x 10^6^ | at 20 d |  |
| Natural Seawater |  | 12:12 | 0.045 | 12.0 x 10^6^ | NA |  |
| CPCC | Glycerol | 12:12 | 0.072 | 12.0 x 10^6^ | NA |  |
| CPCC | Glucose | 12:12 | 0.044 | 10.0 x 10^6^ | NA |  |
| CPCC |  | 12:12 | 0.046 | 12.0 x 10^6^ | NA |  |

**Supplemental Table 2:**  Summary of strains producing anti-CD20, Herceptin, and Rituxan antibodies in shake plate or shake flask. Engineering column describes genetic changes made to the strains. PP, *P. pastoris*; HP, *H. polymorpha*; KM, *K. marxianus*; AA, *A. adeninivorans*. LT, *L. tarentolae*; TR, *T. reesei*; AO, *A. oryzae*. HC_2A_LC, antibody HC and LC sequences are linked by the 2A linker. HC/LC, HC and LC sequences are split in two DNA constructs and integrated at the same locus by homology recombination. NA, not available. BDL, below detection limit of Octet.

| **Species** | **Antibody** | **Strain** | **Engineering** | **Octet Titer (μg/mL)** | **Full-length antibody secreted** |
| --- | --- | --- | --- | --- | --- |
| PP | None | Y486pp | *yku70Δ;* Prepared for multiplexing | 0.00 ± 0.00 | No |
| PP | Anti-CD20 | Y242 | *aox1Δ::pAOX1>HC_2A_LC, yku70Δ* | 5.40 ± 1.14 | No |
| PP | Anti-CD20 | Y800 | *aox1Δ::pAOX1>HC_2A_LC, yku70Δ, dnl4Δ* | 5.56 ± 0.98 | No |
| PP | Anti-CD20 | Y126 | *aox1Δ::pAOX1>HC_2A_LC; yku70Δ, dnl4Δ, pTDH3>VTH1* | 9.77 ± 0.41 | No |
| PP | Anti-CD20 | Y829 | *aox1Δ::pAOX1>HC_2A_LC, dnl4Δ, pTDH3>VTH1, pTDH3>CNE1, pTDH3>ECM10, pTDH3>ERO1* | 11.17 ± 0.06 | No |
| PP | Herceptin | Y324 | *pep4Δ::pAOX1>HC/LC, aox1Δ, yku70Δ* | 8.67 ± 0.68 | Yes |
| PP | Herceptin | Y676 | *pep4Δ::pAOX1>HC/LC, aox1Δ, yku70Δ, pTDH3>CNE1,* *pTDH3>ECM10, pTDH3>ERO1, pTDH3>VTH1* | 11.95 ± 0.84 | Yes |
| PP | Rituxan | Y328 | *pep4Δ::pAOX1>HC/LC, yku70Δ* | BDL | Yes |
| HP | None | Y578 | *yku80Δ*, Prepared for multiplexing | 0.00 ± 0.00 | No |
| HP | None | Y840 | *yku80Δ, yps1Δ*; Prepared for multiplexing | 0.00 ± 0.00 | No |
| HP | None | Y842 | *yku80Δ, yps1Δ, pep4Δ, prb1Δ;* Prepared for multiplexing | 0.00 ± 0.00 | No |
| HP | Anti-CD20 | Y253 | *gas4Δ::pMOX1>HC/LC, yku80Δ* | 3.60 ± 0.07 | No |
| HP | Anti-CD20 | Y138 | *gas4Δ::pMOX1>HC/LC, och1Δ::pMOX1>HC_2A_LC, yku80Δ* | 5.76 ± 0.26 | No |
| HP | Herceptin | Y021 | *och1Δ::pTDH3>HC/LC^a^, yku80Δ* | NA | Yes |
| HP | Herceptin | Y022 | *och1Δ::pTDH3>HC/LC^a^, yps1Δ, yku80Δ* | NA | Yes |
| HP | Herceptin | Y023 | *och1Δ::pTDH3>HC/LC^a^, yps1Δ, pep4Δ, prb1Δ, yku80Δ* | NA | Yes |
| KM | None | Y366 | *yku70Δ,* Prepared for multiplexing | 0 ± 0.00 | No |
| KM | Anti-CD20 | Y350 | *pep4Δ::pTEF>HC/LC, prb1Δ:: pTEF>HC/LC, mnn4Δ::pGAL1>HC/LC, yku70Δ* | NA | No |
| KM | Herceptin | Y486km | *yku80Δ ::pTEF>HC/LC, yku70Δ* | 7.26 ± 0.71^b^ | Yes |
| KM | Herceptin | Y487 | *prb1Δ ::pTEF>HC/LC, yku70Δ* | 2.52 ± 0.16^b^ | Yes |
| KM | Herceptin | Y631 | *yku80Δ ::pTEF>HC/LC, pep4Δ ::pTEF>HC/LC, yku70Δ* | 12.39 ± 0.70^b^ | Yes |
| KM | Herceptin | Y629 | *yku80Δ ::pTEF>HC/LC, pep4Δ ::pTEF>HC/LC,*  *prb1Δ ::pTEF>HC/LC, yku70Δ* | 17.88 ± 0.47^b^ | Yes |
| KM | Rituxan | Y543 | *prb1Δ ::pTEF>HC/LC, yku70Δ* | NA | Yes |
| AA | None | Y244 | Prepared for multiplexing | 0.00 ± 0.00 | No |
| AA | Anti-CD20 | Y412aa | *lys2Δ::pILV1>HC/LC* | 10.75 ± 0.50 | No |
| AA | Anti-CD20 | Y060 | *yku70Δ::pILV1>HC/LC,*  *lys2 Δ* | 10.65 ± 0.35 | No |
| AA | Herceptin | Y858 | *lys2Δ::pILV1>HC/pENO1>LC* | NA | Yes |
| LT | Anti-CD20 | Y396 | *odcΔ::*pT7-*HC_2A_LC* | 3.9^c^ | Yes |
| LT | Herceptin | Y935 | *odcΔ::*pT7-*HC_2A_LC* | NA | Yes |
| TR | Anti-CD20 | Y385 | *pCBH1>CBH1_HC/CBH1_LC* | NA | Yes |
| AO | Anti-CD20 | Y960 | *pGlaA>GlaA_HC/GlaA_LC* | 1.3^c^ | Yes |
| AO | Herceptin | Y976 | *pGlaA>GlaA_HC/GlaA_LC* | NA | Yes |

1. Both HC and LC were fused to *S. cerevisiae* invertase secretion tag.
2. Titers were achieved using the cell recycle shake plate model. See Materials and Methods.
3. Shake flask data with single replicate.

**Supplemental Table 3:**  Maximum growth rates and peak cell densities achieved in *L. tarentolae* cultures while eliminating components of animal origin. At time of inoculation, 5 μg/mL of hemin or 500 μg/mL of catalase was included in the media. At 48 hours, 0.3 μg/mL of hemin or 30 μg/mL of catalase was spiked into the cultures. Growth was followed up to 96 hours. Growth rates measure from 40-48 hours.

| **Media Base** | **Additives** | **μ_max_  (h^-1^, at 40-48 h)** | **Peak Cell Density (cells per mL)** |
| --- | --- | --- | --- |
| BHI | Hemin | 0.070 | 61 x 10^6^ at 72 h |
| BHI | Hemin spike | 0.070 | 74 x 10^6^ at 72 h |
| YE | 1% Fructose + Hemin | 0.074 | 56 x 10^6^ at 64 h |
| YE | 1% Fructose + Hemin spike | 0.073 | 77 x 10^6^ at 64 h |
| YE | 1% Fructose + Catalase | 0.041 | 25 x 10^6^ at 64 h |
| YE | 1% Fructose + Catalase spike | 0.041 | 24 x 10^6^ at 64 h |
| YE | 1% Glucose + Hemin | 0.053 | 59 x 10^6^ at 64 h |
| YE | 1% Glucose + Hemin spike | 0.055 | 71 x 10^6^ at 64 h |
| YE | 1% Glucose + Catalase | 0.054 | 33 x 10^6^ at 48 h |
| YE | 1% Glucose + Catalase spike | 0.057 | 41 x 10^6^ at 64 h |

**Supplemental Table 4:** Herceptin expressing strains constructed in *H. polymorpha*. PA, *S. cerevisiae* pre-alpha secretion tag. IV, *S. cerevisiae* invertase secretion tag. Supernatant samples were loaded on the Western blot in Supplemental Figure 5.

| **Sample** | **Description** |
| --- | --- |
| 1 | Wild type *H. polymorpha* strain (Y578) |
| 2 | *gas4Δ*::*pMOX1*>HC/LC with PA secretion tag |
| 3 | *gas4Δ*::*pMOX1*>HC/LC with IV secretion tag |
| 4 | *och1Δ*::*pMOX1*> HC/LC with IV secretion tag |
| 5 | *och1Δ*::*pTDH3*> HC/LC with IV secretion tag (Y021) |
| 6 | Wild type *H. polymorpha* with *yps1Δ* (Y840) |
| 7 | *gas4Δ*::*pMOX1*> HC/LC with PA secretion tag + *yps1Δ* |
| 8 | *och1Δ*::*pMOX1*> HC/LC with IV secretion tag + *yps1Δ* |
| 9 | *och1Δ*::*pTDH3*> HC/LC with IV secretion tag + *yps1Δ* (Y022) |
| 10 | *H. polymorpha* strain with *yps1Δ* *pep4Δ prb1Δ* (Y842) |
| 11 | *gas4Δ*::*pMOX1*> HC/LC with PA secretion tag + *yps1Δ pep4Δ prb1Δ* |
| 12 | *och1Δ*::*pMOX1*> HC/LC with IV secretion tag + *yps1Δ pep4Δ prb1Δ* |
| 13 | *och1Δ*::*pTDH3*> HC/LC with IV secretion tag + *yps1Δ pep4Δ prb1Δ* (Y023) |

**LIST OF SUPPLEMENTAL FIGURES**

Supplemental Figure 1: Growth Data

A: Average Optical Density at 600 nm of *K. marxianus* Y366 (green circle), *A. adeninivorans* Y244 (purple open square), *H. polymorpha* Y578 (orange triangle), and *P. pastoris* Y486pp (blue open diamond) after 48 hours in YP feedstock (fructose, glucose, glycerol, hydrolysate, lactose, and sucrose) with various carbon sources at 4% concentration. Error bars represent ±1 standard deviation. N = 4. B: Some illumination is necessary for heterotrophic growth of *P. tricornutum.* Average cell density (cells mL^-1^) of *P. tricornutum* cultures in heterotrophic and mixotrophic conditions is plotted over time. Arrows represent a cell recycle and increase in illumination event: (1) cell recycle and illumination increase from no light to 1:23 cycle (1-hour light, 23 hours dark); (2) cell recycle and illumination increase from 1:23 cycle to 12:12 cycle. Media used in this experiment are CPCC F/2 synthetic seawater (purple) supplemented with 1% glucose (orange), 1% glucose and 1% ammonium chloride (blue), 1% glycerol (black), 1% glycerol and 1% ammonium chloride (green), or 1% ammonium chloride (yellow). N=12. Error bars ±1 standard deviation. C. *L. tarentolae* growth achieved on animal-free media. Average cell density (cells mL^-1^) of *L. tarentolae* cultures is plotted over time in YE media (1% glucose) supplemented with 0 μg/mL of hemin (orange), 0.5 μg/mL of hemin (green), 300 (black), and 400 (blue) μg/mL fungal derived purified catalase enzyme or 400 (purple) and 600 (yellow) μg/mL horseradish derived purified peroxidase enzyme. N=8 wells per condition.

A:


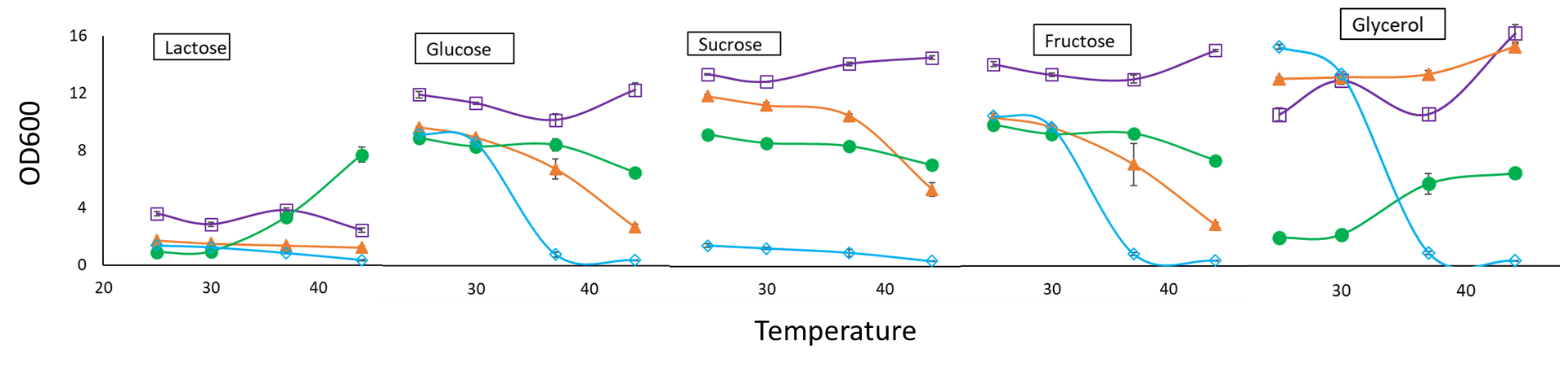


B: C:


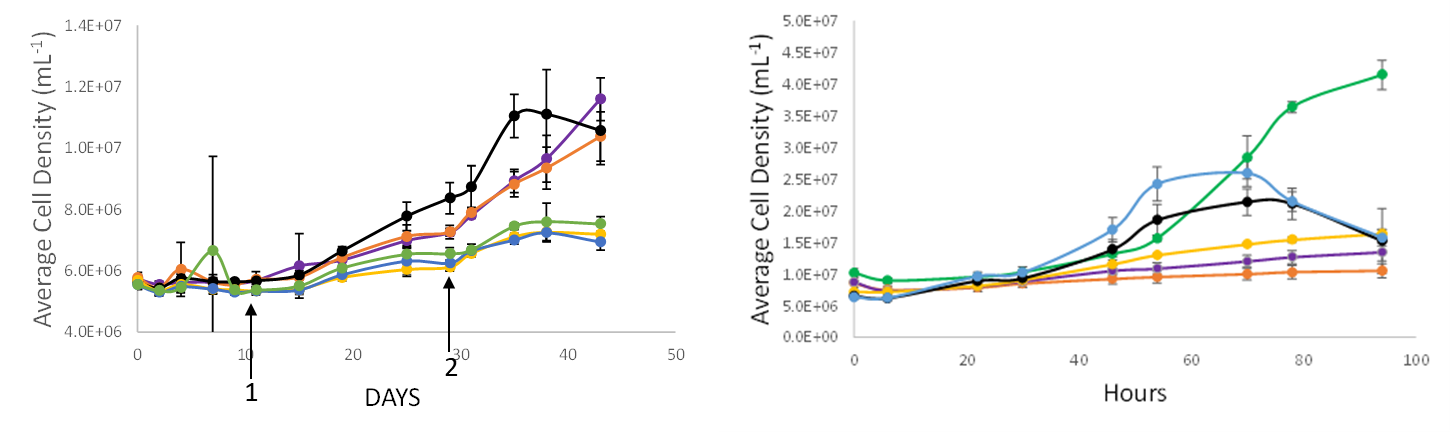


**Supplemental Figure 2:**  Intra-, but not extracellular protease activity rapidly degrades purified Anti-CD20 antibody. Purified Anti-CD20 standard antibody (Std) was incubated with “spent media” or lysates from wild type strains (+/- heat-kill) and sampled at the indicated time points (0 and 21 hours) for analysis on a Western blot using an HC+LC primary antibody for detection. Standard Anti-CD20 antibody sample incubated in PBS (Std in PBS) was used as a control. PP, *P. pastoris*; HP, *H. polymorpha*; KM, *K. marxianus*; AA, *A. adeninivorans*. UT, un-treated cell lysate; HK, heat-killed cell lysate. Supe, supernatant samples. Sc protease Δ, a *Saccharomyces cerevisiae* strain with protease genes *pep4*, *prb1*, and *bar1* deleted. MW, molecular weight marker.


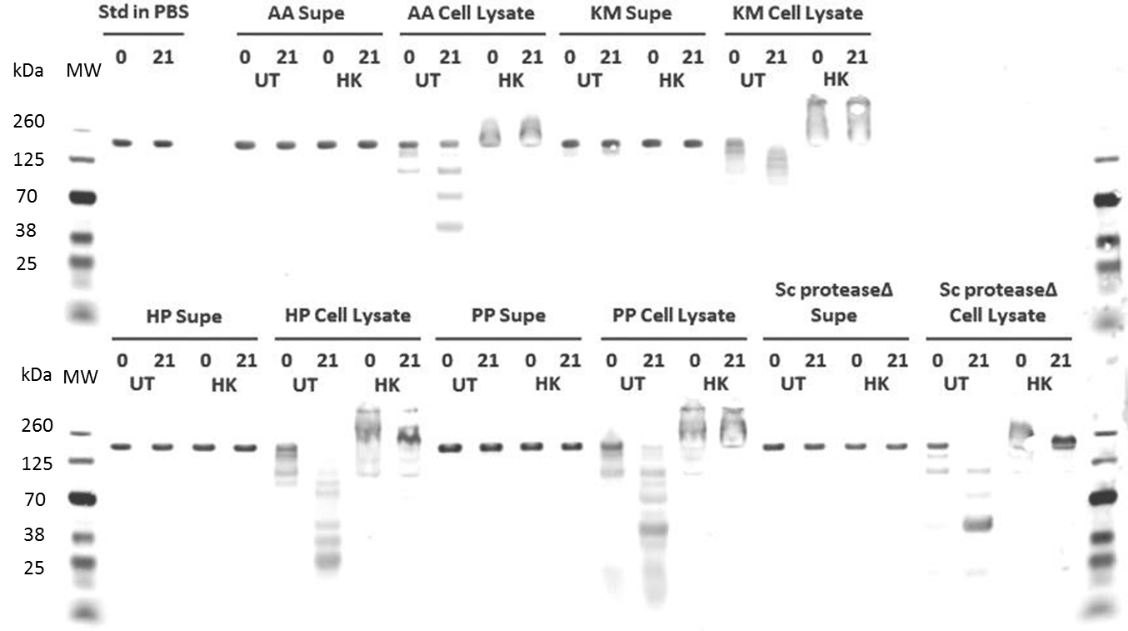


**Supplemental Figure 3:**  Secretion of full-length Herceptin and Rituxan by engineered *P. pastoris* strains. Protein A purified samples were assayed by Western blot under non-reducing (A) and reducing (B) conditions. 1-4, Protein A purified samples; 5-8, Protein A purified and Endo H_f_ treated samples. 1 and 5, Herceptin with pre-alpha secretion leader sequence; 2 and 6, Herceptin with pre-alpha secretion leader sequence and KR mutated to TR in LC; 3 and 7, Rituxan with Kar2 leader sequence; 4 and 8, Rituxan with Kar2 sequence and KR mutated to TR in LC. MW, molecular weight marker, with quantities on the left of each gel. +, Anti-CD20 antibody standard.


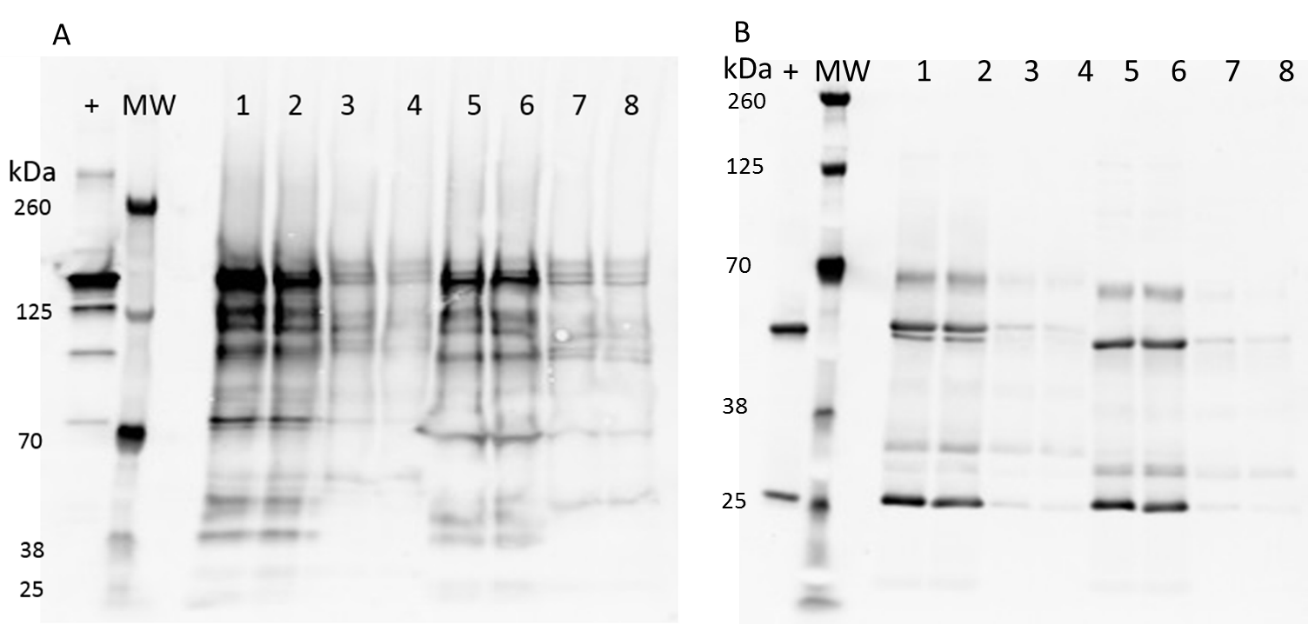


**Supplemental Figure 4:**  Secretion of full-length Herceptin and Rituxan by engineered *K. marxianus* strains. Western blot analysis was performed after growing cells in a 25 mL culture media in a flask at 30°C using cell recycle (lanes 1-6) or standard process (lane7-12). SDS-PAGE gels were run under non-reducing (A and B) or reducing (C) conditions. All strains were grown using YP media cane syrup (4% sucrose). Samples in the non-reducing (B) and reducing condition (C) were Endo H_f_ treated. Lanes 1 and 7 – wild type strain Y366; lanes 2 and 8 – one copy of Herceptin integrated at *YKU80*, Y486km; lanes 3 and 9 – one copy of Rituxan, Y543; lanes 4 and 10 – one copy of Herceptin integrated at *PRB1*, Y487; lanes 5 and 11 – two copies of Herceptin, Y631; and lanes 6 and 12 – three copies of Herceptin, Y629. Refer to Supplemental Table 2 for details on the strains. +, Anti-CD20 standard. MW, molecular weight marker.


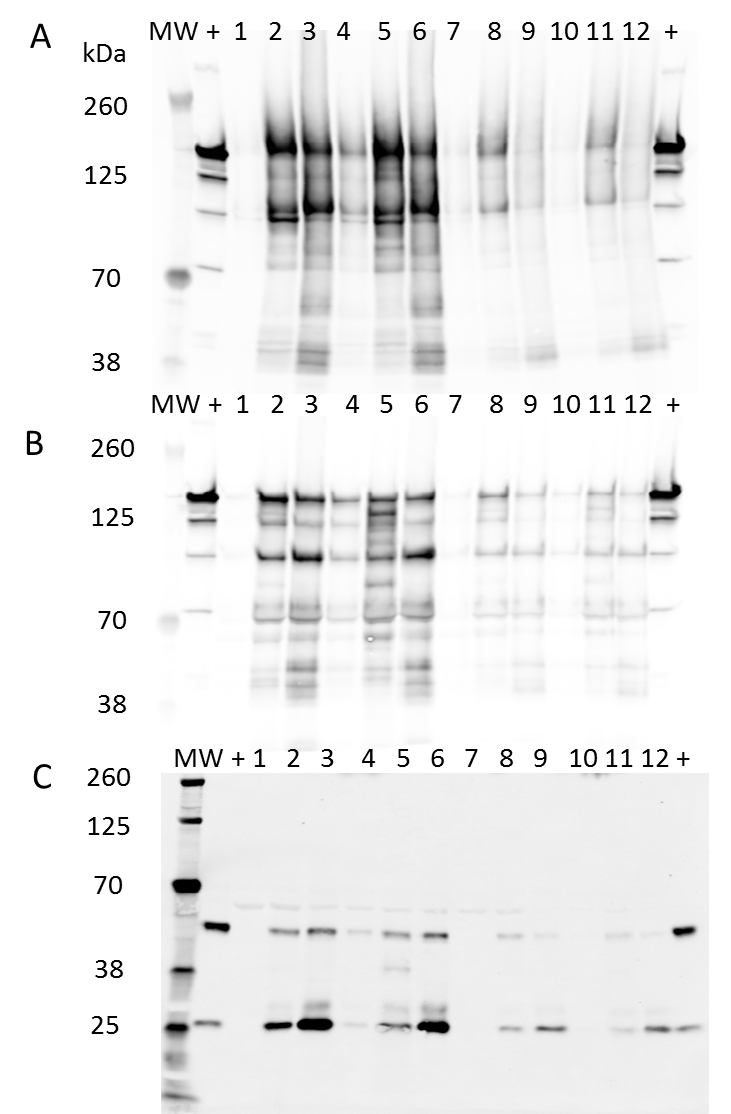


**Supplemental Figure 5:**  Secretion of full-length Herceptin by engineered *H. polymorpha* strains. Western blot analysis of Herceptin expressing strains. (A), Western blot performed under non-reducing conditions. (B), Western blot performed under reducing conditions. Supernatants were harvested from strains grown in production flasks (for *pMOX1* strains - YP 2% methanol + 0.5% glycerol; for *pTDH3* strains - YP 2% glucose) for 48 hours at 30°C. All samples were Protein A purified and Endo H_f_ glycosidase treated prior to Western blots analysis. Strains are described in Supplemental Table 4. +, Anti-CD20 antibody standard; MW, molecular weight ladder. Arrows in (A) indicate full-length antibody. The upper arrow indicates heavy chain and the lower arrow indicates light chain in (B).


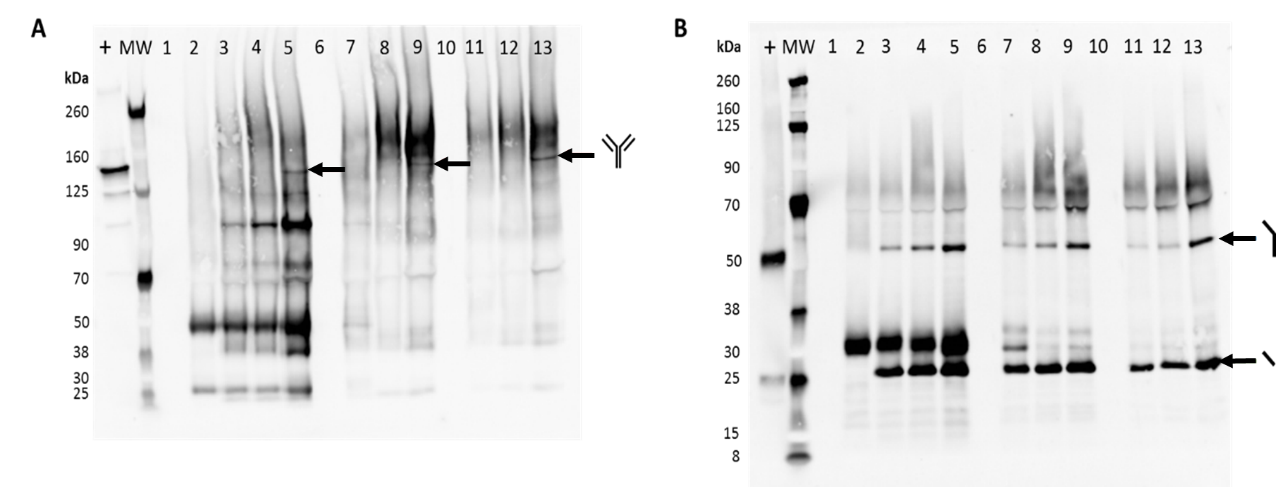


**Supplemental Figure 6:**  Characterization of *in vitro* anti-CD20 antibody degradation by spent *A. oryzae* and *T. reesei* production media as a function of time and protease inhibitor addition. Purified anti-CD20 antibody was spiked into spent media from *A. oryzae* (A) or *T. reesei* (B) and samples were assayed by Western blot at 0, 2.5, and 18-hour time points. Testing was conducted with and without protease inhibitors. Selected inhibitors were added to the spent media and allowed to incubate for approximately five minutes before the addition of the anti-CD20 antibody.


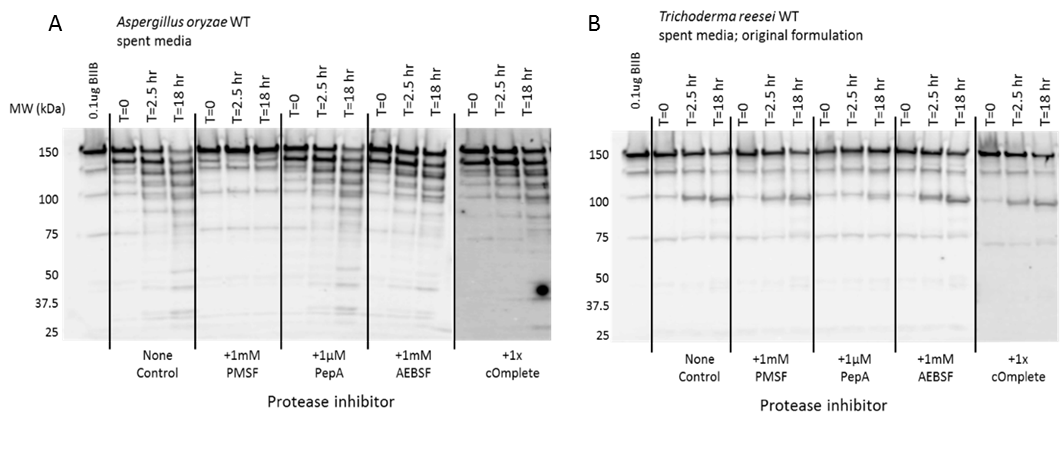

Supplement: Supplementary file 1 — Supplementary information [file BIT-116-1449-s001.docx]
